# Supplementary material for: Circulating Tumor Cells in Head and Neck Squamous-Cell Carcinoma Exhibit Distinct Properties Based on Targeted Epithelial-Related Markers
Source: Curr Issues Mol Biol. 2025 Mar 29;47(4):240. doi: 10.3390/cimb47040240 (PMC12025473; doi:10.3390/cimb47040240)
Supplement: Supplementary file 1 [file cimb-47-00240-s001.zip › cimb-3539479-supplementary.pdf]

Table S1. List of PCR primers used in this study.

Gene name	Assay ID
EPCAM	Hs00158980_m1
EGFR	Hs01076090_m1
MET	Hs01565576_m1
VIM	Hs00958111_m1
CDH1	Hs01023895_m1
CDH2	Hs00983056_m1
SNAI1	Hs00195591_m1
ZEB1	Hs00232783_m1
ZEB2	Hs00207691_m1
TWIST1	Hs00361186_m1
CD274	Hs01125301_m1
PDCD1LG2	Hs01057777_m1
ACTB	Hs01060665_g1
